# Effect of cellulosic fibres on the interfacial friction between ceramic and glass

**DOI:** 10.1038/s41598-025-30490-8

**Published:** 2025-12-08

**Authors:** Javier Rodriguez-Rodriguez, Richard Greenwood, Lewis Taylor, Jonathan Phipps, David Skuse, Zhenyu Zhang

**Affiliations:** 1 School of Chemical Engineering, University of Birmingham, Birmingham, B15 2TT UK; 2 Par Moor Centre, FiberLean Technologies, Par Moor Road, Par, PL24 2SQ UK; 3 Present Address: Par Moor Centre, IMERYS, Par Moor Road, Par, PL24 2SQ UK

**Keywords:** Cellulose fibres, Grinding media, Wear tracks, Lubricant, Shear-thinning, Soft materials, Colloids, Organic molecules in materials science, Chemical engineering

## Abstract

The effect of Micro-Fibrillated Cellulose (MFC) on the interfacial friction between ceramic and glass was investigated as a function of the applied load, surface roughness, sliding distance, sliding velocity, and liquid present. With a normal load of 4 N, high friction was observed whilst maintaining a low wear. Increasing the roughness (*R*_a_) of the ceramic substrate from 0.99 to 3.01 µm could result in a slight increase in the Coefficient of Friction (CoF) from 0.36 to 0.46, but a remarkable increase in wear (depth of the wear track) from 0.27 to 1.31 µm. Friction and wear thresholds were identified after a sliding distance of 400 mm, whilst an increased sliding velocity could reduce both friction and wear. This study also investigated MFC with Xanthan Gum (XG), both with similar rheological behaviour, confirming that the physical presence of fibres in the formulation was responsible for the improved interfacial lubrication.

## Introduction

A wide range of materials and strategies have been investigated to reduce interfacial friction and wear^1–3^, highlighting the use of nanoscopic objects as lubricant additives, which involve the dispersion of nanoparticles within a liquid to enhance the tribological performance of the contact interface^4,5^. Of these materials, the commonly used ones include nanoparticles made of metals, metal oxides, sulphides, carbon-based, or nanocomposites^5–7^. Another long-standing strategy is the incorporation of organic friction modifiers (OFMs), essential additives, in oils for appropriate engineering applications. OFMs could reduce friction through their amphiphilic nature—they could self-assemble on the solid surfaces, forming vertically oriented and closely packed monolayers^8^. Cyriac and colleagues^9^ reported that reagent-grade amphiphilic molecules used as OFMs could effectively reduce friction under typical internal-combustion engine conditions, including heavy loads, low sliding speeds, and elevated temperatures.

The proposed mechanisms by which surface additives reduce interfacial friction and wear include: (a) *rolling*, where particles act as ball bearings between surfaces^9^; (b) *protective film* formation, where particles create an amorphous layer on the surface^10^; (c) *mending*, the lubricants accumulate on the rubbing surfaces^11^; and (d) *polishing*, where the particles used smoothen the surface^3,12,13^. Another aspect contributing to the reduction of friction and wear is the morphological characteristics of the lubricant additives used. For instance, carbon nanotubes, due to their anisotropic nanostructure, length, rigidity, and high aspect ratio, could effectively enhance interfacial friction^14^. Other combinations of nanodiamonds with organic and inorganic friction modifiers enhanced tribofilm formation reducing friction under elevated temperatures^15^. Similarly, organic fibres, particularly cellulose fibres, might exhibit comparable behaviour when in a liquid environment. As an example, recent studies have shown that cellulose nanocrystals used in biolubricants can reduce friction by up to 44%, likely due to stable film formation^16^, as well as graphene-cellulose nanoparticle formulations have presented tribological improvements within the contact interface^17^.

Cellulose molecule is a linear polymer consisting of *D*-glucopyranose ring units linked by *β*-1,4-glycosidic bonds^18,19^. Hydrogen bonding within the chain arranges the elementary fibrils into bundles known as microfibres^20^. Cellulose fibres, on the other hand, possess a cylindrical hollow structure called lumen, encased by a cell wall^18,21^. Such hierarchical structure, combined with its mechanical and chemical properties, offer cellulose fibres a potential to act as a bio-based lubricating additive^22^. Additionally, cellulose fibres can present different shapes and dimensions, such as Micro-Fibrillated Cellulose (MFC) that is produced by breaking the cell wall, forming a three-dimensional network of microfibres^23,24^.

To investigate the interfacial lubrication properties of MFC, ceramic and glass were selected as the solid substrates in contact. The tribological characteristics of ceramics and glass has been extensively investigated under various parameters, including normal load, surface roughness, sliding distance, velocity, and lubricated scenarios^25,26^. It is hypothesised that the effectiveness of MFC in improving the interfacial lubrication is determined not only by its structural and mechanical properties but also on its viscosity^27^. Recent studies demonstrated the possibility of using MFC fibres as eco-friendly lubricants to reduce wear and heat in various processes by preventing the direct contact between solid substrates^22,28,29^. Ilyin and co-workers^30^ reported that using MFC as a thickener in triethyl citrate oil could reduce interfacial friction and wear at low shear rates, which was attributed to an increased viscosity and the interaction between microfibres and surfaces, forming a thin tribological film. In contrast, Blok and colleagues^31^ reported that mayonnaise thickened with MFC exhibited the highest Coefficient of Friction (CoF) compared to other thickeners such as starch, waxy corn starch, and Xanthan gum (XG), suggesting that the microfibre aggregates could hindered the formation of a lubrication film. There have been contrast views on the role of MFC in interfacial friction, highlighting the need for a systematic investigation.

This work investigated the tribological characteristics of MFC fibres at room temperature between different combinations of solid substrates. A systematic investigation of the tribological conditions was carried out, including the material of the media beads, normal force, sliding velocity, sliding distance, and substrate roughness. Additionally, different concentrations of MFC were compared with XG to assess the effect of rheological properties^32^ of the tribological characteristics.

## Materials and methods

### Material

Ceramic and glass beads (Body 1), along with Micro-Fibrillated Cellulose (MFC) slurry, were kindly donated by FiberLean Technologies. Two types of solid substrates (Body 2), glass slides (DELTALAB, Spain) and alumina substrates with varied roughness (RS Components Ltd, UK) were used in the friction experiments. Various dilutions of the MFC slurry were prepared to achieve suspensions with 0.07%, 0.69%, and 2.82% fibre content. Xanthan Gum (XG) purchased from Special Ingredients Ltd, UK, was used to prepare solutions of varied concentrations of 0.25% and 4.50% in matching the shear viscosities of the MFC suspensions at 0.69% and 2.82% fibre content.

### Tribological measurements

Friction experiments were conducted using a friction tester (ForceBoard, Industrial Dynamics AB, Sweden) equipped with horizontal and tangential load cells. A single bead was glued to a screw that is attached to the arm of the equipment. The arm executes a reciprocating sliding motion of the bead against the substrate under various conditions outlined in Table 1. The friction tester employs a full 2D force sensing, converting mechanical load into voltage signals that are amplified and proportional to the applied load in N. Tangential (friction force) and normal forces were continuously measured and recorded using the DAQ Factory software. The CoF in the second steady state was calculated as the ratio of the friction force (*F*) to the applied load (*L*), represented as *μ* = *F*/*L*.

**Table 1:** Testing parameters used for the tribological experiments.

Parameters	Values
Beads	Ceramic (Young’s modulus of 150 GPa) and glass (Young’s modulus of 70 GPa) beads of 3.00 mm diameter
Normal load (N)	1–7
Sliding distance (mm)	50–1600
Sliding velocity (mm/s)	1, 4 and 20
Substrates	Glass, Alumina A (*R*_a_ 1.00 µm), Alumina B (*R*_a_ 2.20 µm), and Alumina C (*R*_a_ 3.00 µm)
Lubricants	Water; MFC suspension (0.07, 0.69, and 2.82 wt%); XG aqueous solution (0.25 and 4.50 wt%)

**Fig. 1:**
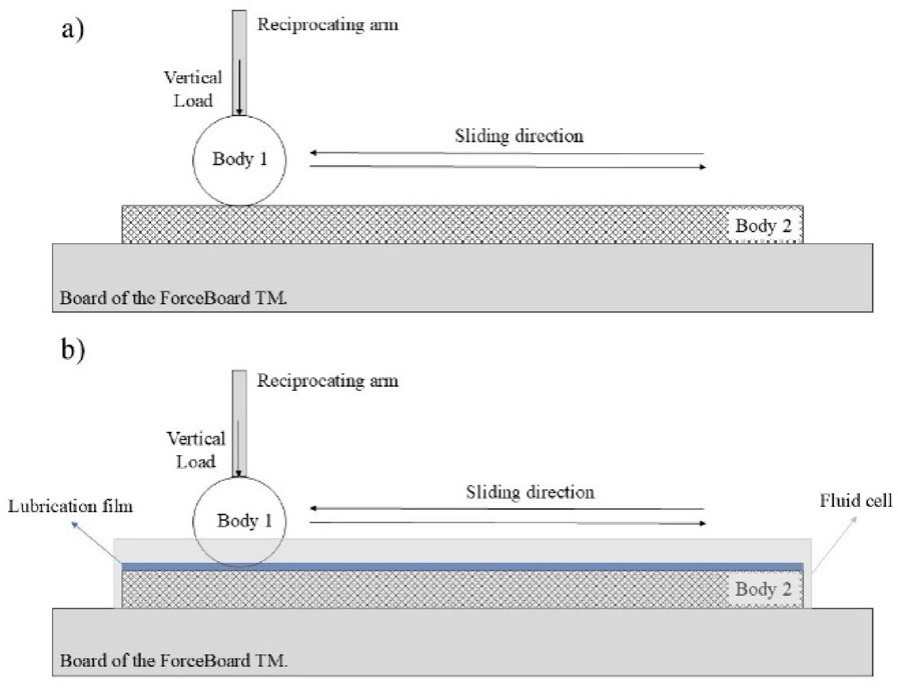
Schematic of the ForceBoard (Industrial dynamics AB, Sweden) with the elements of the tests for (**a**) ambient condition and (**b**) aqueous environment.

Figure 1 presents the schematic diagram of the frictional measurements under two scenarios: (a) ambient environment and (b) liquid environment with the presence of MFC or XG. A custom fluid cell (36 × 96 mm) was designed, and 3D printed to accommodate the glass substrates (25 × 75 mm) enabling a lubricant layer of approximately 2 mm thick. This configuration ensured sufficient lubricant entrainment during the experiments.Each tribological test consisted of 30 cycles, performed in triplicate at 20 °C (the interest in this study remains at low temperature). Beads (Body 1) and substrates (Body 2) were replaced after each replicate to minimise the wear effect on the system. Contact pressure was calculated using Hertzian theory, considering the apparent area of contact with parameters supplied by the supplier^33^.

For the effect of roughness, the contact pressure was calculated with the real area of contact using *TriboSolver*^34^ that applies the Boundary Element Method (BEM), using a half-space theory approximation to solve an integral system shown in Eq. 1^34–39^.

1 $$\left\{ {\begin{array}{*{20}l} {h_{e} \left( {x,y} \right) = \frac{2\pi }{{E^{\prime } }}\iint {\frac{{p\left( {x^{\prime } ,y^{\prime } } \right)}}{{\left( {x - x^{\prime } } \right)^{2} + \left( {y - y^{\prime } } \right)^{2} }}dx^{\prime } dy^{\prime } }} \hfill \\ {h_{e} \left( {x,y} \right) = z\left( {x,y} \right) - \delta ,\forall \left( {x,y} \right) \in A_{c} } \hfill \\ {p\left( {x,y} \right) > 0,\forall \left( {x,y} \right) \in A_{c} } \hfill \\ {F_{N} = \iint {p\left( {x^{\prime } ,y^{\prime } } \right)dxdy}} \hfill \\ \end{array} } \right\}$$

where *h*_e_ is the elastic deflection, 1/*E*′ the reduced elastic modulus, *p(x,y)* the contact pressure, *z(x,y)* the surface roughness profile, *δ* the rigid body approach, *A*_C_ the contact area, and *F*_N_ the applied normal load.

Table 1 summarises all parameters used in the tribological experiments. For the study of roughness effects, three alumina substrates characterised by different surface roughness values (*R*_a_ ranging from 1 to 3 µm) were used. Glass beads were not tested in this part of the study. Some experiments involved lubrication scenarios for which a fluid cell was designed and 3D printed using polylactic acid (PLA, Verbatim). The fluid cell facilitated the formation of a lubricant film between the two counter-bodies (Body 1 and Body 2).

### Surface morphology characterisation

**Fig. 2:**
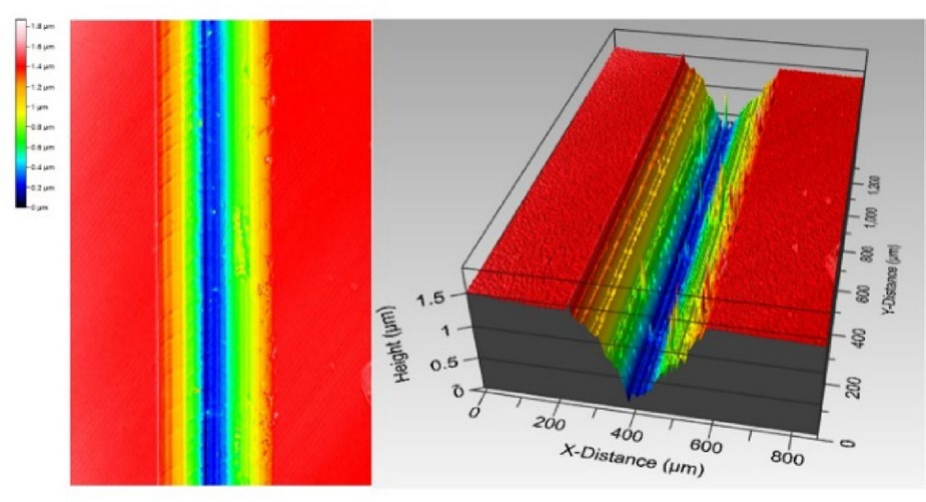
A representative wear track of the glass bead across the glass substrate, acquired by WLI.

Surface roughness and wear track were evaluated using a White Light Interferometer (WLI) (MicroXAM2, Omniscan), with analysis performed using the Scanning Probe Image Processor (SPIP). Averaged surface roughness (*R*_a_) and root mean square (*R*_q_) values were used to characterise substrate roughness. Measurements were taken from three different locations on each sample. Wear depth was determined by measuring and averaging the depth profile along the wear track (y direction, see Fig. 2) on three samples. Mean depths in the y direction were calculated and compared across samples.

### Rheological evaluation

Rheological measurements were performed using a hybrid rheometer Discovery HR-1 from TA Instruments with a cup and bob geometry. The shear viscosity was measured in triplicate at 20 °C as a function of increasing shear rate from 1 to 1100 s^−1^.

## Results and discussion

### Surface topography of the solids in contact

**Fig. 3:**
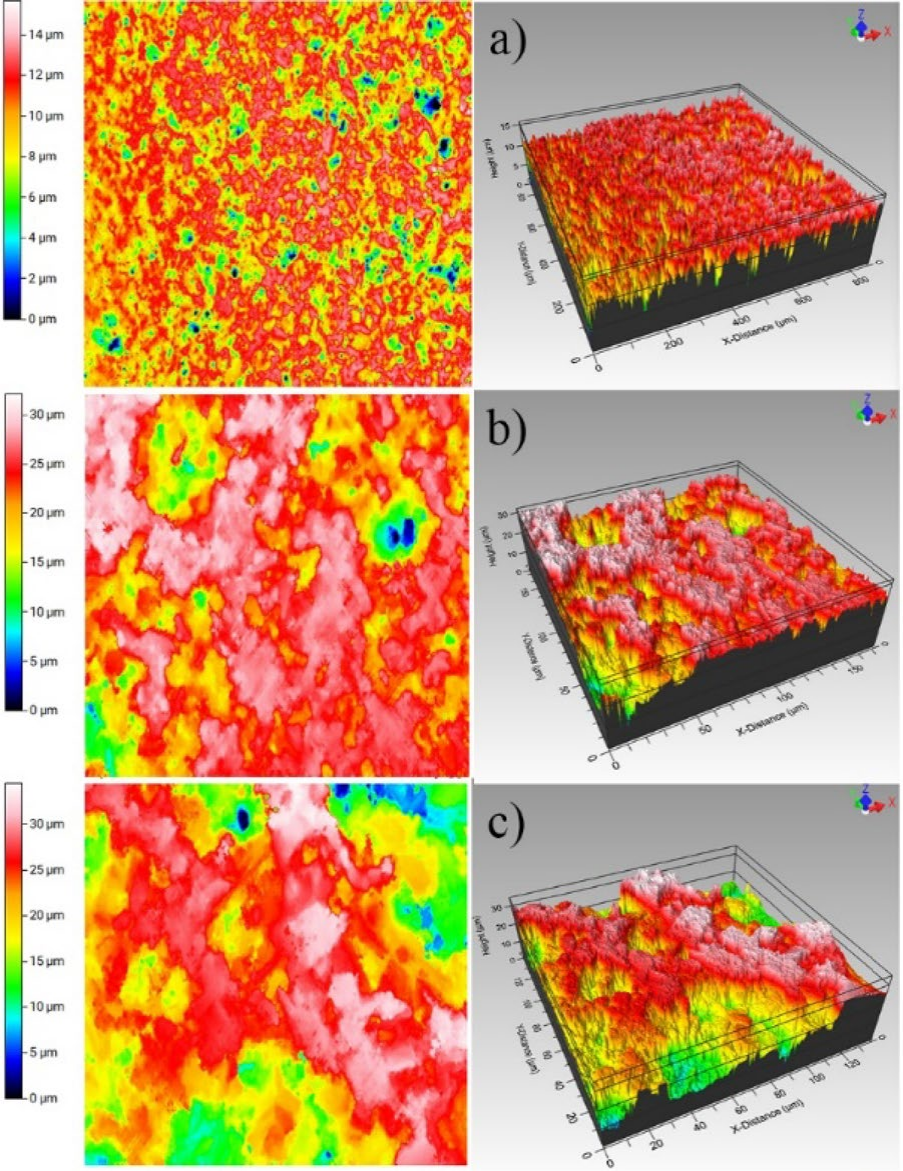
2D (left column) and 3D (right column) profilometry images for (**a**) Alumina A, (**b**) Alumina B and (**c**) Alumina C.

WLI measurements were carried out to obtain surface topography of the solids in contact, from which two surface roughness parameters: *R*_a_ and *R*_q_, were extracted. Representative topography images of three alumina substrates are shown in Fig. 3. For Alumina A, the surface roughness values are *R*_a_ 1.00 µm and *R*_q_ 1.31 µm. For Alumina B, *R*_a_ 2.20 µm and *R*_q_ 2.56 µm, and for Alumina C, *R*_a_ 3.00 µm and *R*_q_ 3.51 µm. The differences in roughness can be clearly observed in Fig. 3, and are in concordance with the values reported in the literature^40^. Surface roughness results provide vital information with regards the contact geometry and the corresponding contact area.

### Rheological profile of MFC and XG suspensions

**Fig. 4:**
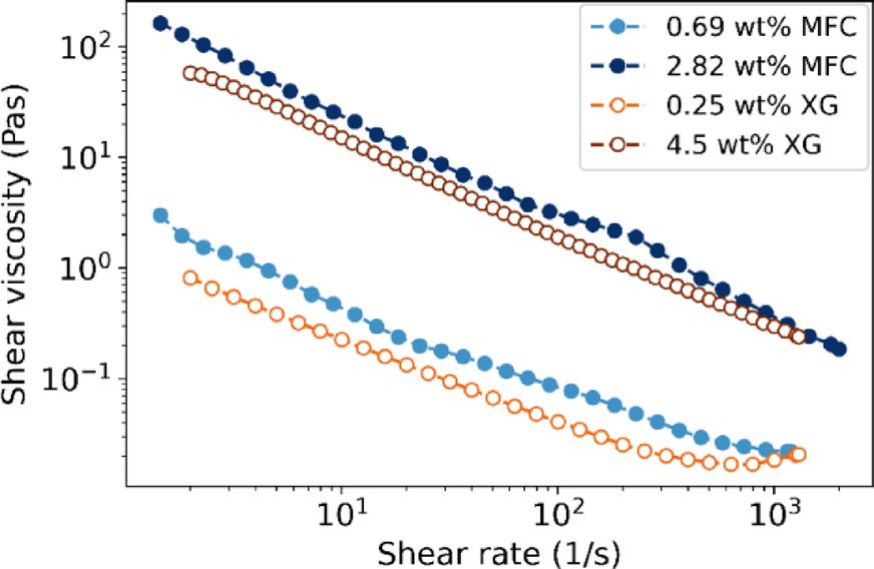
Shear viscosity versus shear rate for MFC slurry and XG.

To elucidate the lubrication mechanism of the MFC suspension, XG solutions were selected to produce liquid that matches the specific rheological characteristics of MFC used. With its shear-thinning characteristics, XG is an appropriate candidate based on some previous work by Zhong and colleagues^41^. A calibration curve using different concentrations of XG was constructed to match the viscosities of MFC suspensions at a reference shear rate of 1100 s^-1^. Figure 4 shows flow curves for MFC slurries (0.69% and 2.82% fibre content) and XG (0.25% and 4.50%), evidencing the similarity between the suspensions, which is consistent with the finding reported by Blok and colleagues^32^. It confirms that both MFC and XG exhibit consistent rheological behaviour.

### Tribological characteristics

For a frictional experiment, a CoF is established upon the initial contact (known as run-in period), during which the counter-bodies would experience a gradual topographical change and wear^42^. The surface asperities make contacts, resulting in an increased CoF^43^, following which the CoF reaches a steady-state. We would like to highlight that the reported CoF values in this study correspond to the averaged CoF during the steady-state phase obtained in these experiments over a sliding distance ranging from 400 to 1000 mm (ensuring that the run-in period had concluded). Wear tracks were observed on the substrates due to contact, and were characterised by the averaged depth along the track direction of three different samples. The reported results represent the mean value obtained from these three measurements.

#### Effect of load on the normal direction under ambient

**Fig. 5:**
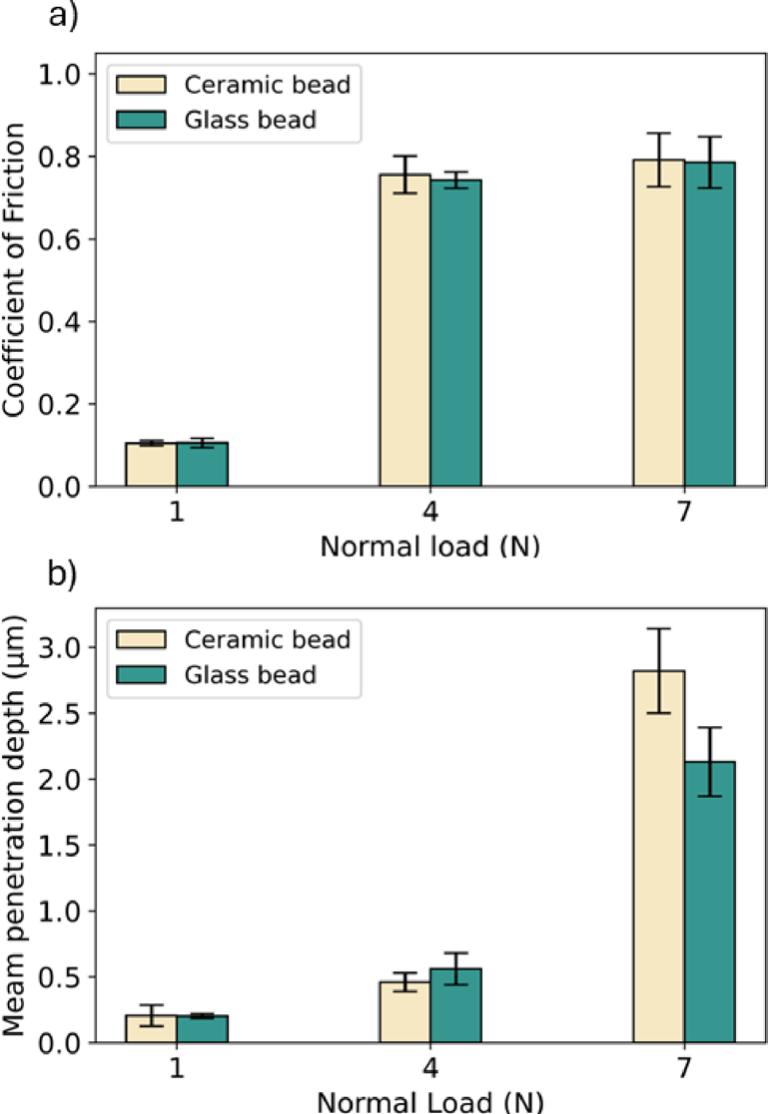
Variation of (**a**) Coefficient of Friction and (**b**) mean depth of the wear track as a function of the normal load for ceramic and glass beads (ambient condition, sliding velocity = 2 mm/s, sliding distance = 200 mm, 30 loops) on glass substrate.

Figure 5 shows the effect of normal load on friction and wear under the ambient environment without the presence of MFC or XG solutions. Figure 5a shows that applying normal loads around 1 N (correspond to contact pressure of 349 MPa for ceramic and 292 MPa for glass) results in small CoF values, 0.10 for both ceramic and glass beads. With an increased normal load, e.g. 4 N (corresponding contact pressure of 553 MPa for ceramic, 463 MPa for glass) and 7 N (corresponding contact pressure of 667 MPa for ceramic, 558 MPa for glass), the CoF values were found to increase considerably to 0.76, 0.74 and 0.79, 0.78, respectively, which is likely due to the changed surface topography. Applying 4 N of normal load is not large enough to change the surface morphology, since Fig. 5b shows low penetration depths. The surface asperities deform and fracture when the applied load is above 4 N, leading to an increased contact area and consequently enhanced CoF values.

Hwang and coworkers^44^ tested different composites using an applied load ranging from 10 to 50 N with a similar tribometer, and they attributed the increased CoF with vertical load to the increased contact area, which is consistent with our observation. Likewise, Ming and colleagues^45^ tested an advanced ceramic under water lubrication, and postulated that the increase in CoF with vertical load from 1 to 14 N was due to the change of tribological contact from hydrodynamic lubrication to boundary lubrication where the surfaces conformed, increasing both the contact area and CoF. Figure 5b presents the mean depth measured post the tribological tests of varying normal loads. For ceramic beads, the depth ranged from 206 nm to 2.82 µm, and for glass beads, from 202 nm to 2.13 µm. It can be concluded that high normal loads resulted in an increased CoF and wear under the ambient environment without the presence of MFC or XG, which is likely due to the changes in the tribological contact.

#### Effect of surface roughness under ambient

**Fig. 6:**
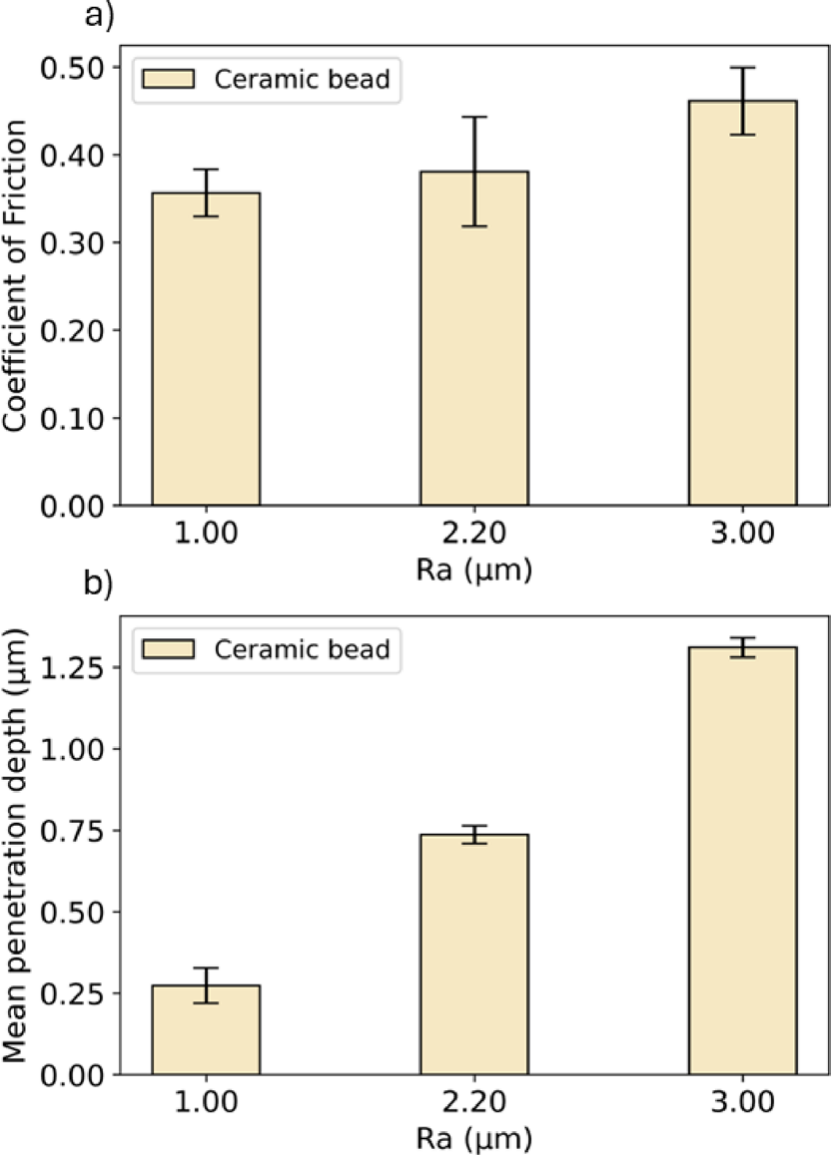
(**a**) Coefficient of Friction and (**b**) mean depth of the wear track as a function of surface roughness (*R*_a_) for ceramic bead (ambient condition, normal load = 5 N, sliding velocity = 2 mm/s, sliding distance = 200 mm, 30 loops) on the alumina substrates.

To evaluate the effect of surface roughness on the tribological characteristics, three alumina substrates of varied roughness (*R*_a_ = 1.00, 2.20, and 3.00 µm) were prepared. Figure 6 presents the CoF values and wear under the ambient condition, using a ceramic bead, without MFC or XG solution. With an applied normal load of 5 N, the contact pressure was 594, 767, and 829 MPa for *R*_a_ values of 1.00, 2.20, and 3.00 µm, respectively. Figure 6a shows that CoF increases from 0.36 at *R*_a_ = 1.00 µm to 0.46 at *R*_a_ = 3.00 µm. And a similar trend was observed for wear: Fig. 6b reveals that the mean wear track depth increases from 0.27 to 1.31 µm, suggesting a correlation between CoF and wear. Based on Persson’s theory^42^, the real contact area, thus the friction and wear, is determined by the power spectrum of the surface roughness. As surface roughness increases, the apparent contact area and real contact area decrease, concentrating the contact pressure on the high asperities on the surfaces. This results in an increased localised pressures and a large degree of deformation of these asperities during sliding, leading to an increased friction and wear^46,47^. The values of CoF and wear for the alumina substrates increase with surface roughness, which is consistent with Persson’s predictions. The observation agrees with the results reported by Al-Samaraie et al.^48^ who used a pin-on-disk tribological rig to demonstrate an increased friction and wear with the samples of the highest surface roughness. Kato^49^ also reviewed the role of surface roughness on metallic alloys and ceramic, highlighting that surface roughness had the strongest effect on wear.

#### Effect of sliding distance under ambient

**Fig. 7:**
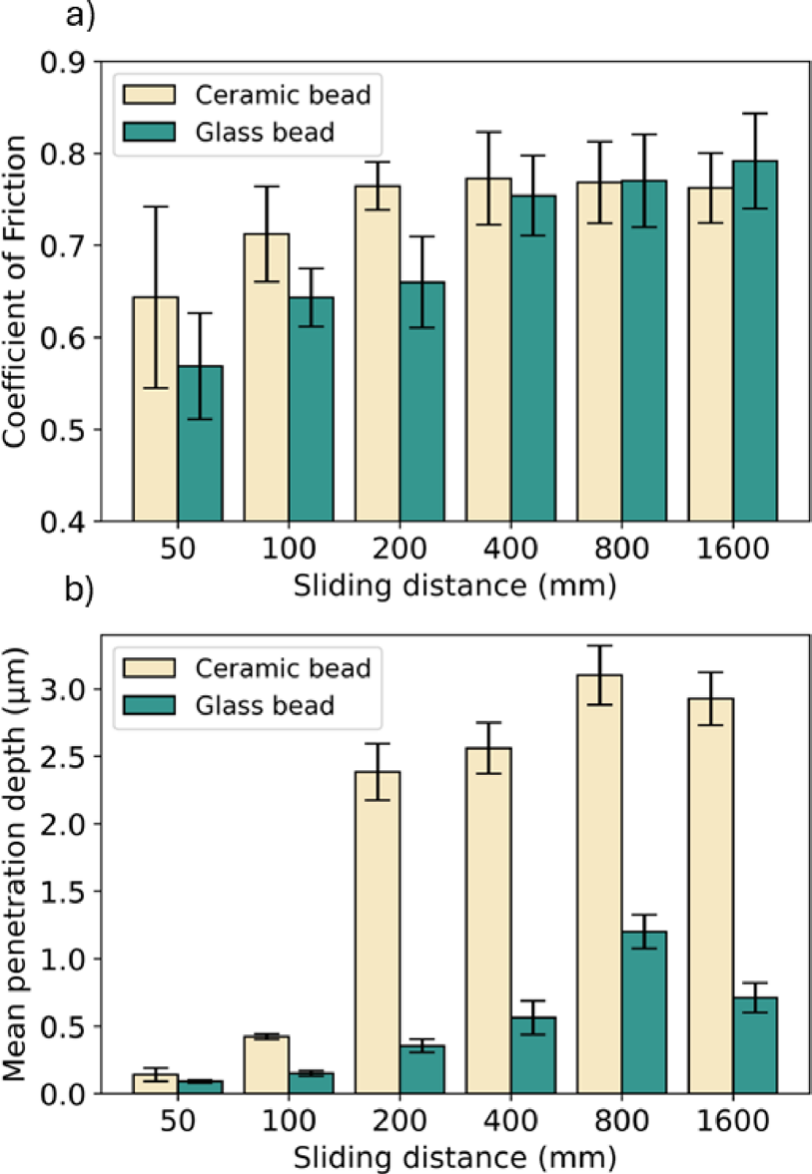
Variation of (**a**) Coefficient of friction and (**b**) mean depth of the wear track with the variation of sliding distance for ceramic and glass beads (ambient friction, normal load = 5 N, sliding velocity = 2 mm/s, 30 loops) on glass substrate.

Figure 7 presents the influence of sliding distance on CoF and wear for ceramic and glass beads, which could advance our understanding of the evolving wear mechanisms. Figure 7a shows that CoF values increase with the sliding distance, but reaches a plateau after 200 mm for ceramic and 400 mm for glass beads. Ceramic beads show greater CoF values than glass beads, which is likely because ceramic, as a material, has a greater hardness than glass. Figure 7b presents that an increased wear track was observed for both materials with a prolonged sliding distance, which is likely due to the increased contact time. The drastically increased penetration depth, from 100 to 200 mm, is likely because of the accumulation of wear debris on the indenter, which accelerates the wear at the contact^50^. It is worth noting that the wear track depth varies significantly between ceramic and glass due to the differences in the hardness between them, presenting a maximum penetration depth of 3.10 µm for ceramic and 1.2 µm for glass bead. The behaviour in the micro-contacts are determined by the mechanical properties of the asperities^51,52^, whereby ceramic bead has a lower elasticity but greater hardness than glass bead, resulting in a more notable wear to the glass substrate^46,53^. Comparing this effect to the results in Fig. 5, at a normal load of 7 N for 200 mm, the difference in wear track between both materials is reduced, which suggests that the effect of mechanical properties on lubrication and wear is no longer viable when the normal load is adequately. For large sliding distances, the CoF for ceramic reaches a threshold, implying that the wear reaches a steady state, which is consistent with previous studies on ceramic substrates^54–56^.

#### Effect of sliding velocity under ambient

**Fig. 8:**
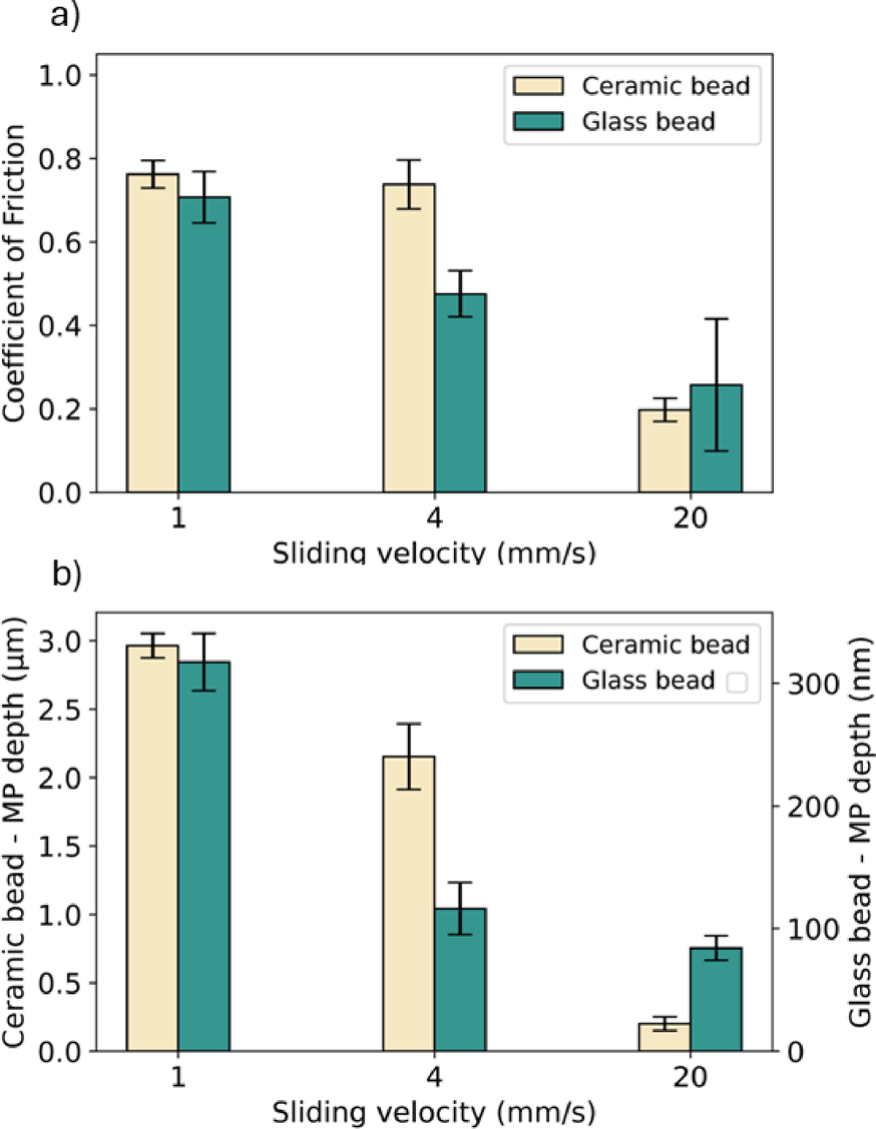
(**a**) Coefficient of Friction and (**b**) mean depth of the wear track (MP depth) for ceramic and glass beads (ambient friction, normal load = 5 N, sliding distance = 200 mm, 30 loops) on glass substrate.

Finally, to establish a comprehensive understanding of the tribological contact and to construct a Stribeck curve, the effect of sliding velocity on CoF and wear for ceramic and glass beads was measured under the ambient condition (Fig. 8). Figure 8a shows that CoF decreases with an increasing sliding velocity for glass beads, reducing from 0.70 (1 mm/s) to 0.26 (20 mm/s). For the first two values of sliding velocity (1 and 4 mm/s), ceramic beads exhibit very similar CoF (~ 0.75), which decreases to 0.19 at 20 mm/s. Figure 8b shows the mean depth of the wear track, which decreases with an increasing sliding velocity (from 2.96 µm at 1 mm/s to 200 nm at 20 mm/s for ceramic beads, and from 317 to 84 nm for glass beads). The reduction in CoF with sliding velocity suggests that an increasing sliding velocity could reduce the contact at asperity level, thereby reducing the frictional resistance^57^.

#### Effect of MFC and its concentration

**Fig. 9:**
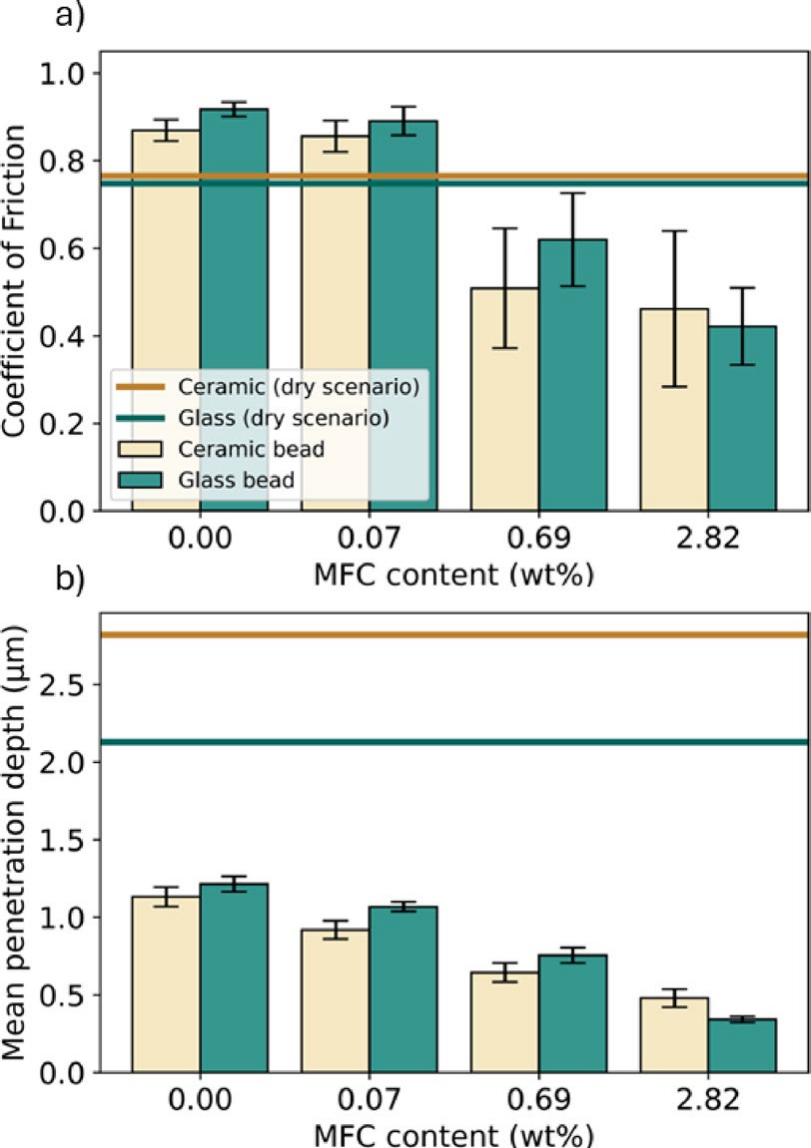
The influence of MFC fibre content on (**a**) Coefficient of Friction and (**b**) mean depth of the wear track for ceramic and glass beads on glass substrate. (normal load = 5 N, sliding velocity = 2 mm/s, sliding distance = 20 mm, 30 loops).

The effect of MFC and XG on the interfacial friction under aqueous condition was evaluated using the same measurement method under the normal load of 5 N, sliding velocity of 2 mm/s, and sliding distance of 20 mm. Figure 9a shows CoF values acquired, evidencing that MFC suspensions of varied fibre contents could considerably reduce the interfacial CoF, in comparison to the CoF values acquired in ambient condition or water only. Both ceramic and glass beads exhibit slightly greater CoF values with water and low MFC content (0.07 wt%) than those in ambient conditions. However, the CoF values decrease by 45% when the MFC content was increased to 0.69 wt% and 2.82 wt%. Figure 9b presents the mean depth of the wear track post the friction measurements, showing a drastically reduced wear once MFC was introduced in the suspension, in contrast to the wear tracks acquired in ambient environment. While it is evidenced that an increasing MFC content in the suspension would result in a reduced wear track depth for both ceramic and glass beads, the improvements become less pronounced beyond 0.69 wt%, indicating that 0.69 wt% may already be adequate for forming a robust load-bearing MFC network in the contact area.

It is worthwhile to highlight that great CoF values when water or 0.07 wt% MFC suspension was introduced was unexpected, as water-lubricated scenarios normally result in lower friction in comparison with dry friction. WLI images of the wear tracks revealed that, in the two scenarios mentioned, the scratches exhibited a more defined shape, and therefore higher contact. This might lead to an increased adhesive interaction at the contact, which explains the greater frictional force observed than that in the ambient. Additionally, the presence of water could mobilise wear debris within the contact, causing these particles to act as a third body that increase friction and widen the scratches. Obtaining the mean depth of the wear tracks was convenient and reproducible, however, with a change in the shape is less accurate to measure.

The significantly reduced CoF when using MFC required some additional measurements to verify our hypothesis that fibres are the responsible for the interfacial lubrication. After identifying the XG solutions that match the rheological properties of MFC slurry at 0.69 and 2.82 wt% (Fig. 4), frictional experiments were carried out using both ceramic and glass beads.

**Fig. 10:**
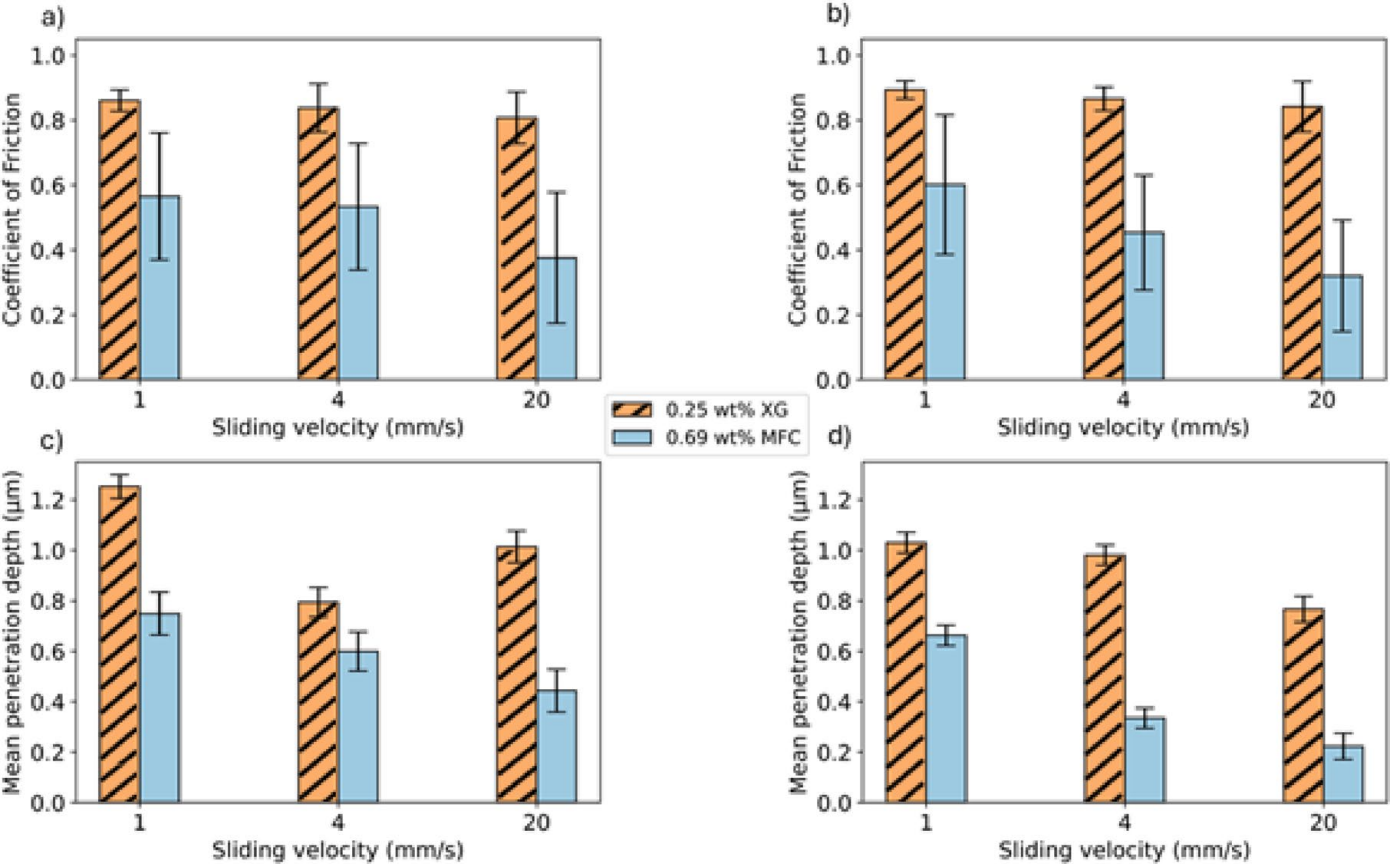
Coefficient of friction (**a**) for ceramic beads and (**b**) for glass beads against sliding velocities at 0.69 wt% MFC and 0.25 wt% xanthan gam (normal load = 5 N, sliding distance = 20 mm, 30 loops) on glass substrate. The resulting mean depth of the wear track (**c**) for ceramic beads and (**d**) for glass beads with the sliding velocity at 0.69 wt%MFC and 0.25 wt% xanthan gam (normal load = 5 N, sliding distance = 20 mm, 30 loops) on glass substrate.

The comparison between MFC and XG is presented in Fig. 10 (XG = 0.25 wt% XG and MFC = 0.69 wt%), whereby Fig. 10a and b illustrate the friction results for ceramic and glass, respectively. The CoF values were found in the region of 0.8 with the presence of the XG solutions, regardless the sliding velocity, for both ceramic and glass beads. The data suggests that MFC slurries manifest a marked reduction in CoF compared to XG under the identical measurement conditions, and that an increased fibre concentration correlates with a decrease in CoF values for the MFC suspension. For ceramic beads at 1 mm/s, XG presents a CoF of 0.86, whereas MFC suspension of similar viscosity gives a CoF of 0.56. The effect of MFC suspension was observed consistently in other frictional tests, which provides a compelling evidence that the inclusion of cellulosic fibres leads to lower CoF values while maintaining an equivalent rheological property.

According to the well-established Stribeck curve, increasing the sliding speed could be beneficial in reducing CoF in the mixed lubrication regime. However, the sliding velocity within the range of tribological condition investigated in the present work shows a negligible effect on CoF for XG, regardless of the bead material. This implies that the contact interface studied here likely falls in the boundary lubrication regime whereby friction is predominantly governed by the characteristics of the counter-bodies. Conversely, with the MFC suspension, a significant reduction in CoF was observed as sliding velocity increases. For ceramic beads using MFC, raising the sliding velocity from 1 to 20 mm/s results in a CoF decrease from 0.57 to 0.38. Similarly, for glass beads using MFC, the CoF decreases from 0.60 to 0.32 over the same velocity range. This tribological behaviour supports the hypothesis that the increased gap between the two surfaces facilitates better incorporation of fibres into surface grooves and irregularities. Studies by Kim and Sasaki^58,59^ on similar velocities showed that an increased sliding speed would result in lower CoF values. High sliding velocities examined by Yahiaoui et al. and Xing et al.^60,61^ confirmed this observation, too. Scaraggi and Persson^62^, using different lubricants and based on the effective elastic modulus, offered the same explanation that the sliding friction increases as velocity decreases.

Figure 10c presents the mean depth for ceramic, while Fig. 10d focuses on glass beads. The data clearly demonstrate that the depth profiles vary between XG and MFC. For ceramic and glass, MFC scenarios produce significantly shallower depths compared to XG. These tests reinforce our hypothesis that cellulosic fibres present at the contact interface could significantly impact the friction and wear results observed in this study.

**Fig. 11:**
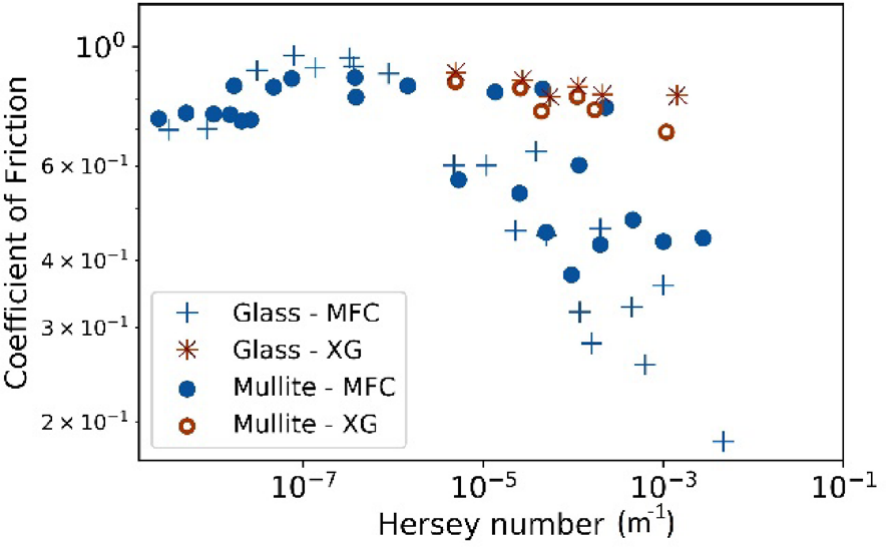
Stribeck curve for ceramic and glass bead at MFC and xanthan gum (sliding distance = 20 mm, 30 loops) on glass substrate.

All results acquired in the present work are summarised to generate a Stribeck curve for MFC and XG and both bead types (Fig. 11), whereby CoF is presented as a function of the Hersey number that is the product of viscosity (N s m^−2^) and sliding velocity (m s^−1^), divided by the normal load (N). The constructed Stribeck curve evidences that XG solution operates within the boundary lubrication regime, indicated by a plateau across all Hersey number values tested. In contrast, the MFC suspension initially shows a plateau, suggesting a boundary regime at low Hersey numbers. However, as the Hersey number increases, CoF values decline, suggesting a transition towards a mixed lubrication regime for both ceramic and glass beads. Based on the friction and wear results, we conclude that the presence of MFC fibres is able to inhibit direct contact between the two solid substrates, reducing the contact between surface asperities. It is very probable these microfibres become trapped within asperities, aiding interfacial lubrication.

## Conclusions

The effect of MFC particles on the interfacial friction between ceramic or glass beads and glass or alumina substrates were studied using a force plate setup. The tribological parameters investigated include the vertical load, substrate roughness, sliding distance, and sliding velocity. The following conclusions can be drawn from the experimental results:

- Under ambient condition, increasing the normal load results in an increased friction (from 0.1 to 0.79 CoF values) and wear (wear track depth from 0.20 to 2.82 µm). The intermediate normal load exhibited a high friction while maintaining a low wear rate. Hence, an optimal normal load could be identified where high friction is achieved while keeping wear at the minimal. Increasing the normal load to 7 N showed no differences in wear results between ceramic and glass, confirming that normal load has a more significant effect on the wear process than the other parameters, such as sliding distance.
- Under the ambient condition, increasing surface roughness led to an increased friction and wear, with the increase in wear being more pronounced than that in friction (from 0.2 to 1.91 µm for wear, while CoF values varied from 0.36 to 0.46).
- As the sliding distance increases, a plateau in both friction and wear was reached, suggesting a damage threshold. Notably, the differences in wear between ceramic and glass are evident, primarily due to the increased hardness of ceramic beads. However, this distinction becomes imperceptible under higher normal load conditions. At the maximum tested normal load of 7 N, the elevated pressure induces a significant damage in both materials, eliminating any discernible difference between them.
- A significant reduction in friction and wear was observed with an increase in sliding velocity, which could have a significant value to the industrial processes where MFC is used.
- The various lubrication scenarios studied demonstrate that MFC fibres play a crucial role in preventing direct contact at the articulating interface. The comparison between MFC and XG explicitly confirms that increasing viscosity within the studied regimes does not necessarily change the tribological characteristics. However, the introduction of MFC shifts the Stribeck curve towards the mixed lubrication regime, resulting in a reduced wear and friction. The finding offers new opportunities of using bio-based cellulosic fibres as potential interfacial active to reduce friction and wear.

## Data Availability

The datasets used and/or analyzed during the current study available from the corresponding author on reasonable request.
